# Evolution of the *SPX* gene family and its role in the response mechanism to low phosphorus stress in self-rooted apple stock

**DOI:** 10.1186/s12864-024-10402-2

**Published:** 2024-05-16

**Authors:** Zenghui Wang, Xiaowen Zhang, Xuemei Yang, Haixia Tang, Lijuan Feng, Yanlei Yin, Jialin Li

**Affiliations:** 1 Shandong Institute of Pomology, Tai’an, 271000 Shandong China; 2 School of Biological Science and Technology, University of Jinan, Jinan, 250022 China

**Keywords:** Self-rooted apple stock, Expression patterns, *SPX* family, Phosphate starvation, Regulatory networks

## Abstract

**Background:**

Phosphorus plays a key role in plant adaptation to adversity and plays a positive role in the yield and quality formation of apples. Genes of the *SPX* domain-containing family are widely involved in the regulation of phosphorus signalling networks. However, the mechanisms controlling phosphorus deficiency are not completely understood in self-rooted apple stock.

**Results:**

In this study, 26 members of the apple *SPX* gene family were identified by genome-wide analysis, and further divided into four subfamilies (*SPX*, *SPX*-*MFS*, *SPX*-*EXS*, and *SPX*-*RING*) based on their structural features. The chromosome distribution and gene duplications of *MdSPXs* were also examined. The promoter regions of *MdSPXs* were enriched for multiple biotic/abiotic stresses, hormone responses and typical P1BS-related elements. Analysis of the expression levels of 26 *MdSPXs* showed that some members were remarkably induced when subjected to low phosphate (Pi) stress, and in particular *MdSPX2*, *MdSPX3*, and *MdPHO1.5* exhibited an intense response to low Pi stress. *MdSPX2* and *MdSPX3* showed significantly divergent expression levels in low Pi sensitive and insensitive apple species. Protein interaction networks were predicted for 26 MdSPX proteins. The interaction of MdPHR1 with MdSPX2, MdSPX3, MdSPX4, and MdSPX6 was demonstrated by yeast two-hybrid assay, suggesting that these proteins might be involved in the Pi-signaling pathway by interacting with MdPHR1.

**Conclusion:**

This research improved the understanding of the apple *SPX* gene family and contribute to future biological studies of *MdSPX* genes in self-rooted apple stock.

**Supplementary Information:**

The online version contains supplementary material available at 10.1186/s12864-024-10402-2.

## Background

Phosphorus (P) is an essential macronutrient for all living organisms. Although phosphorus is abundant in the environment, it is limited due to its low dispersal rate, uptake by the soil, and conversion to organophosphorus by microorganisms. The lack of phosphorus in the soil greatly reduces the growth of plants, and has become the main constraint of agricultural production [1, 2].

Phosphorus plays a key role in plant adaptation to adversity and plays a positive role in the yield and quality formation of apples [3, 4]. According to the soil nutrient conditions in many apple producing areas, it is found that the apple orchards in China are mostly established in the mountains with barren soil layer, water and nutrient shortage and complex climate Hill field, most of the soil has a very low effective P content [5, 6]. In the process of apple cultivation, the lack of effective phosphorus in the soil will delay the growth of trees, form stiff seedlings, delay flowering and maturity, lead to falling flowers and fruits, hinder sugar transportation, and affect the yield and quality of apples. Good root morphology and physiological characteristics are the basis for the absorption and utilization of phosphorus nutrients and adaptation to soil low phosphorus environment [7]. Therefore, the selection of low phosphorus resistant apple stocks is of great significance for the sustainable development of apples.

The *SPX* gene family involved in many phosphate (Pi) signalling pathways and the SPX domain was first identified in a yeast *gpa1* inhibitor (SYG1), a cyclin-dependent kinase inhibitor (PHO81) and human xenotropic and polytropic retrovirus receptor 1 (XPR1) [8, 9, 18]. Based on the presence of additional domains, SPX proteins can be further classified into four subfamilies: SPX proteins, SPX-EXS proteins, SPX-MFS proteins, and SPX-RING proteins [10–12]. The AtSPXs, CoSPXs, GmSPXs, PeSPXs, BnaSPXs, TaSPXs, OsSPXs, and ZmSPXs have been characterized [13–20]. PHOSPHATE RESPONSE 1 (PHR1) as a key regulator of transcriptional responses to Pi starvation, is a transcription factor with an MYB domain and a coiled-coil domain binding to a P1BS *cis*-element, which was identified in *Arabidopsis thaliana* [21]. The certain SPX domain-containing proteins regulated the transcriptional activity of *PHR1*. Under Pi deficiency, the content of cellular inositol pyrophosphate decreases, releasing PHR1 from the SPX1 and PHR1 complex to bind to the promoter of the *PSI* genes, thereby increasing genes transcription [22–25].

Classifying gene family members in the genome in mining biological problems related to species traits is the first step, laying a solid foundation for subsequent gene function studies and genetic transformation studies. However, the mechanisms controlling phosphorus deficiency are not clear in self-rooted apple stock. In this study, a bioinformatics analysis of *SPX* gene family members was performed in the apple genome, including chromosomal locations, phylogenetic relationships, gene structures, gene duplication, and *cis*-element analysis. We further proved that MdSPX2, MdSPX3, MdSPX4, and MdSPX6 physically interacted with MdPHR1. This study revealed the molecular function of *SPX* gene in apples, which can provide a theoretical basis for the breeding of low phosphorus resistance, and has great significance for the sustainable development of the apple industry.

## Results

### Identification and chromosomal locations of apple *SPX* genes

**Fig. 1:**
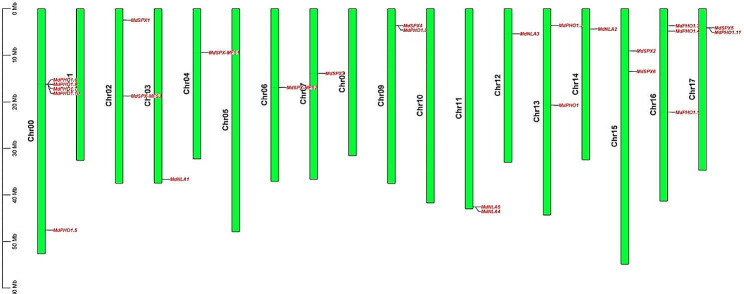
Distribution of *MdSPX* family genes on apple chromosomes

Using 20 *Arabidopsis* SPX proteins as search sequences, a total of 26 apple *SPX* genes were identified by BlastP search in the *Malus×domestica* reference genome database. The accuracy of the apple *SPX* genes was verified based on Pfam database (http://pfam.janelia.org/) and SMART website (http://smart.embl-heidelberg.de/) [26]. The *MdSPX* family genes were named based on their homologs in *Arabidopsis* as follows: six genes belonged to the *SPX* subfamily, the *SPX*-*EXS* subfamily contained 12 genes, three members belonged to the *SPX*-*MFS* subfamily, and five members belonged to the *SPX*-*RING* subfamily. The gene ID, molecular weight, amino acids length and the isoelectric points (pI) of the 26 *MdSPX* genes were summarized in Table 1. The amino acids lengths of the 26 MdSPX proteins ranged from 262aa (*MD07G1115200*) to 835aa (*MD00G1081000*), and the predicted molecular weights varied from 30.21 kD to 96.25 kD. All proteins of these four subfamilies except *MdSPX1*, *MdSPX2*, *MdSPX4*, *MdSPX5*, *MdSPX6*, *MdSPX*-*MFS1*, *MdSPX*-*MFS2*, and *MdNLA3* had a pI greater than seven, indicating that these proteins belonged to basic proteins (Table 1). The chromosomal distribution of the *MdSPX* family genes was shown in Fig. 1. Among the 26 genes in apple, 21 genes were successfully mapped onto 13 chromosomes of apple, and 5 *SPX*-*EXS* subfamily genes were mapped onto Chr0. Chromosomes 3, 4, 6, 7, 12 and 14 contained only one gene, chromosomes 2, 9, 11, 13, 15 and 17 contained two genes, while chromosome 16 contained three genes (Fig. 1).

**Table 1:** Description of *Malus domestica* SPX family genes

Gene ID	Gene name	Location	Molecular weight (kD)	Amino acid length (aa)	pI
*MD02G1031100*	*MdSPX1*	Chr02:2446537–2,449,320	33.12	289	6.20
*MD15G1124700*	*MdSPX2*	Chr15:9047428–9,050,194	33.20	288	5.13
*MD07G1115200*	*MdSPX3*	Chr07:13857569–13,865,493	30.21	262	7.73
*MD09G1054300*	*MdSPX4*	Chr09:3612656–3,615,230	36.91	331	4.89
*MD17G1052000*	*MdSPX5*	Chr17:4091091–4,093,213	41.08	366	5.39
*MD15G1172700*	*MdSPX6*	Chr15:13456527–13,459,263	33.06	289	5.74
*MD13G1216800*	*MdPHO1*	Chr13:20718527–20,723,714	57.78	497	9.27
*MD16G1222100*	*MdPHO1.1*	Chr16:22212605–22,222,240	90.13	778	9.13
*MD13G1049800*	*MdPHO1.2*	Chr13:3542062–3,548,264	92.42	798	9.27
*MD16G1050800*	*MdPHO1.3*	Chr16:3627571–3,632,983	95.03	820	9.22
*MD16G1068000*	*MdPHO1.4*	Chr16:4775222–4,779,764	91.99	799	9.43
*MD00G1200800*	*MdPHO1.5*	Chr00:47531487–47,534,671	80.65	700	9.35
*MD00G1081000*	*MdPHO1.6*	Chr00:16188231–16,191,412	96.25	835	9.36
*MD00G1081300*	*MdPHO1.7*	Chr00:16225026–16,228,305	95.62	829	9.44
*MD09G1054400*	*MdPHO1.8*	Chr09:3615917–3,620,025	90.32	783	9.39
*MD00G1081200*	*MdPHO1.9*	Chr00:16198380–16,203,765	52.47	461	9.21
*MD00G1081400*	*MdPHO1.10*	Chr00:16228664–16,232,575	90.17	778	9.15
*MD17G1052300*	*MdPHO1.11*	Chr17:4116056–4,120,188	89.75	780	9.08
*MD04G1068900*	*MdSPX-MFS1*	Chr04:9395159–9,401,627	78.72	704	6.36
*MD06G1069900*	*MdSPX-MFS2*	Chr06:16904023–16,918,731	75.03	670	6.58
*MD02G1195400*	*MdSPX-MFS3*	Chr02:18714848–18,719,643	74.71	674	7.90
*MD03G1287000*	*MdNLA1*	Chr03:36622212–36,624,655	36.29	316	9.05
*MD14G1045700*	*MdNLA2*	Chr14:4357625–4,361,254	36.07	332	9.15
*MD12G1046800*	*MdNLA3*	Chr12:5372221–5,375,491	38.00	333	6.78
*MD11G1313600*	*MdNLA4*	Chr11:42597648–42,600,287	38.05	316	7.78
*MD11G1313200*	*MdNLA5*	Chr11:42577417–42,579,672	36.19	316	8.88

### Conserved motif and gene structure analysis of *SPX* gene family

**Fig. 2:**
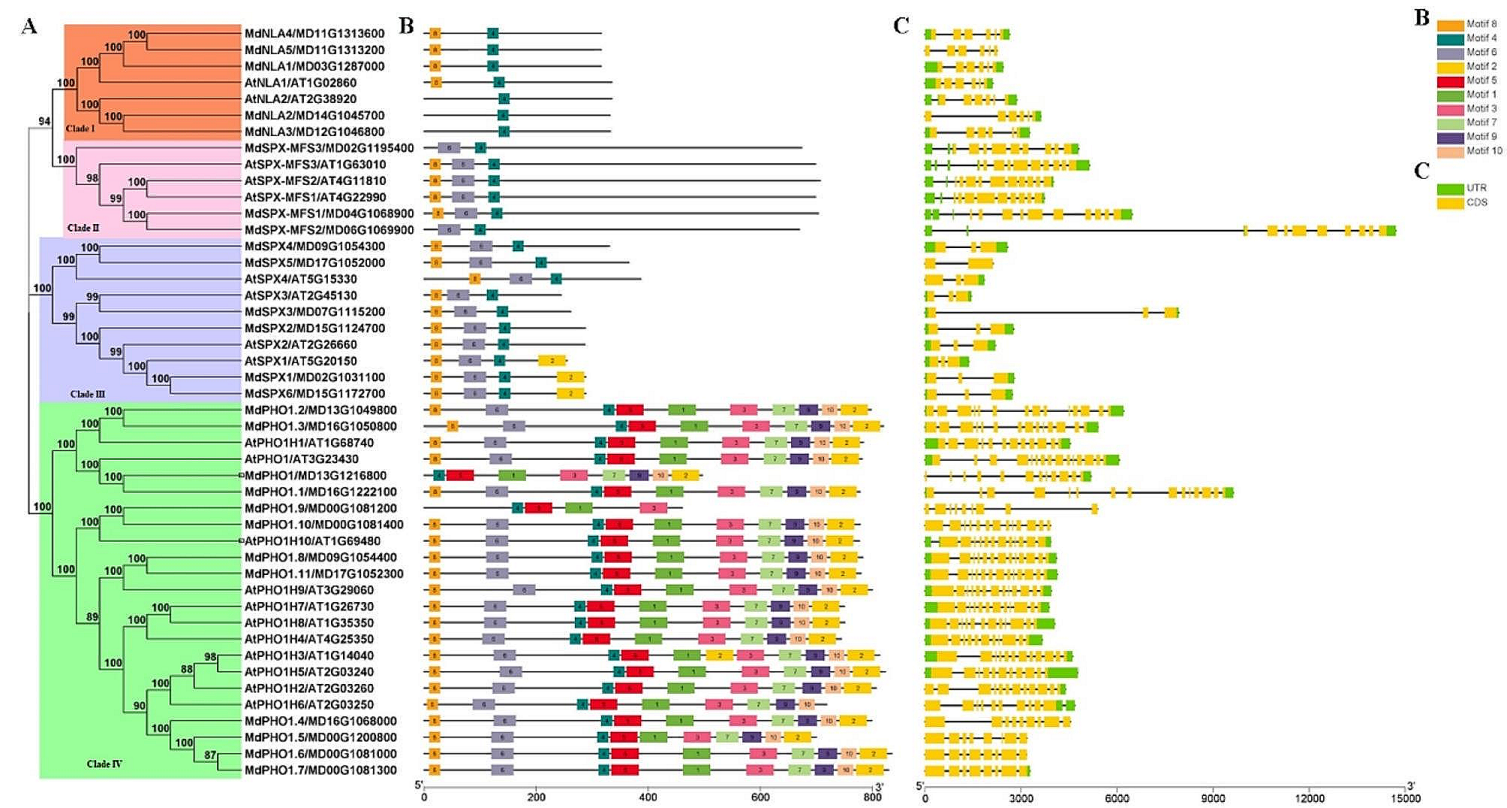
Phylogenetic relationships, conserved motifs and gene structure analysis of apple and *Arabidopsis SPX* genes. (**A**) A phylogenetic tree of 26 MdSPX and 20 AtSPX protein sequences was generated using MEGA 7.0 and clustered into four branches. (**B**) Prediction of conserved motifs in SPX proteins was performed by MEME software. The corresponding 10 conserved sequences were referred to Figure S1. (**C**) Gene structure of *SPXs*.

To preliminarily resolve the phylogenetic relationships of apple SPX proteins, we comparatively analysed the phylogenetic trees, conserved motifs and gene structures of 26 MdSPX and 20 AtSPX in detail (Fig. 2). As shown in Fig. 2A, the phylogenetic tree classified these 26 SPX proteins into four clades, which were named Clade I, Clade II, Clade III, and Clade IV corresponding to the *SPX*-*RING* subfamily, *SPX*-*MFS* subfamily, *SPX* subfamily, and *SPX*-*EXS* subfamily, respectively. Each clade contains *MdSPX* and *AtSPX* genes, and the proteins in the same subfamily were highly related.

For the evolutionary and functional analysis of gene family members, analysing gene structures could provide critical clues. To clarify the exon/intron structure of the *SPX* genes, we visualised their gene structures using GSDS (Fig. 2C). Similar gene structure patterns were presented in the same clade. Of the 26 *SPX* genes, members of the *SPX*-*RING* subfamily contained only six exons, whereas the number of exons ranged from nine to ten in the *SPX*-*MFS* subfamily, eight to fifteen in the *SPX*-*EXS* subfamily, and two to three in the *SPX* subfamily (Fig. 2C).

To further resolve the structural diversity of the SPX proteins and predict their functions, we used MEME to predict the number and composition of conserved motifs in AtSPX and MdSPX proteins (Fig. 2B). Ten different motifs were identified (Fig. S1). Similar motif distribution patterns were found among proteins belonging to the same clade. Motif 4 was present in all SPX proteins, suggesting that this motif was a characteristic motif specific to *SPX* family genes and might be related to the common function of SPX proteins. Some specific motifs were only present in specific clade as follows motifs 1, 3, 5, 7, 9, and 10 were only included in all members of Clade IV subfamily and not in other subfamilies, further confirming the accuracy of the subfamily division. Clade IV was the subfamily with the most motifs, containing 10 motifs, whereas the other three subfamilies contained only 1–3 motifs (Fig. 2B). Thus, the functions of these motifs need to be further investigated to understand how these proteins function.

Overall, the *SPX* genes that were closely related in evolutionary terms in the phylogenetic tree had similar conserved motifs and gene structures, indicating that proteins within the same subfamily may exhibit similar functions.

### Phylogenetic analysis of SPX proteins in different plant species

**Fig. 3:**
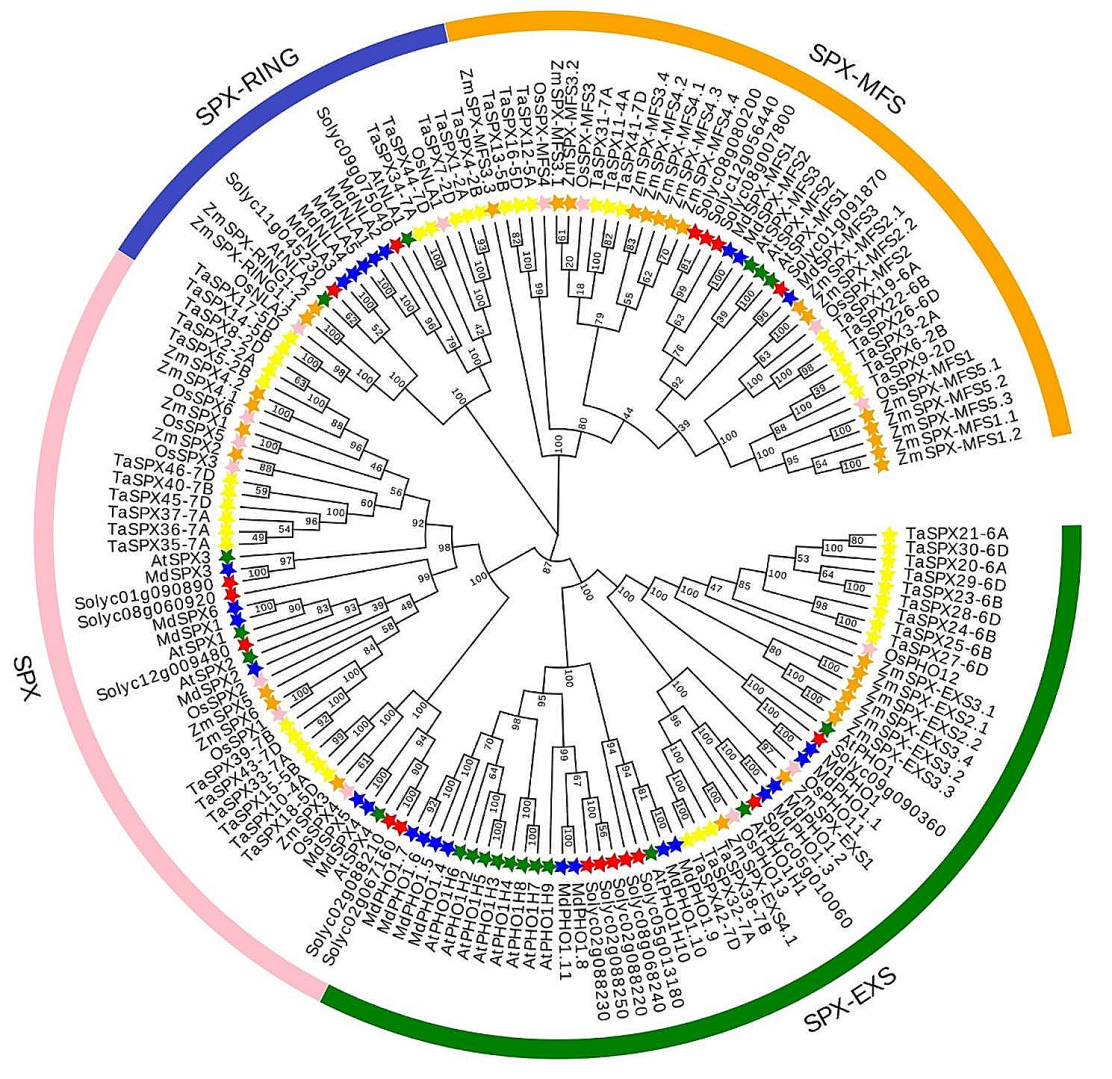
Phylogenetic tree consisting of 157 SPX proteins from *Arabidopsis thaliana*, *Solanum lycopersicum*, *Zea mays*, *Oryza sativa*, *Malus domestica* and *Triticum aestivum*. SPX proteins from different species were tagged with different coloured stars

To further explore the evolutionary relationships of *SPX* gene family members, we performed a CLUSTALW alignment of 157 SPX protein sequences, consisting of 26 MdSPX, 20 AtSPX, 46 TaSPX, 32 ZmSPX, 18 SlSPX, and 15 OsSPX from six different species, and constructed a phylogenetic tree using the NJ method of MEGA 7. These proteins were divided into four subfamilies (Fig. 3), of which *SPX*-*EXS* was the subfamily containing the most proteins. Monocotyledonous (*Triticum aestivum*, *Zea mays*, and *Oryza. Sativa*) and dicotyledonous species (*Solanum lycopersicum*, *Malus domestica*, and *Arabidopsis*) were clearly divided into distinct evolutionary clades. The members of the *SPX*-*EXS* and *SPX*-*MFS* subfamilies showed clear species differentiation, implying that SPX-EXS and SPX-MFS proteins might have different biological functions in distinct plants. In general, phylogenetic analysis could provide reference for the evolution and function of family genes.

### Gene duplication analysis of *MdSPXs*

**Fig. 4:**
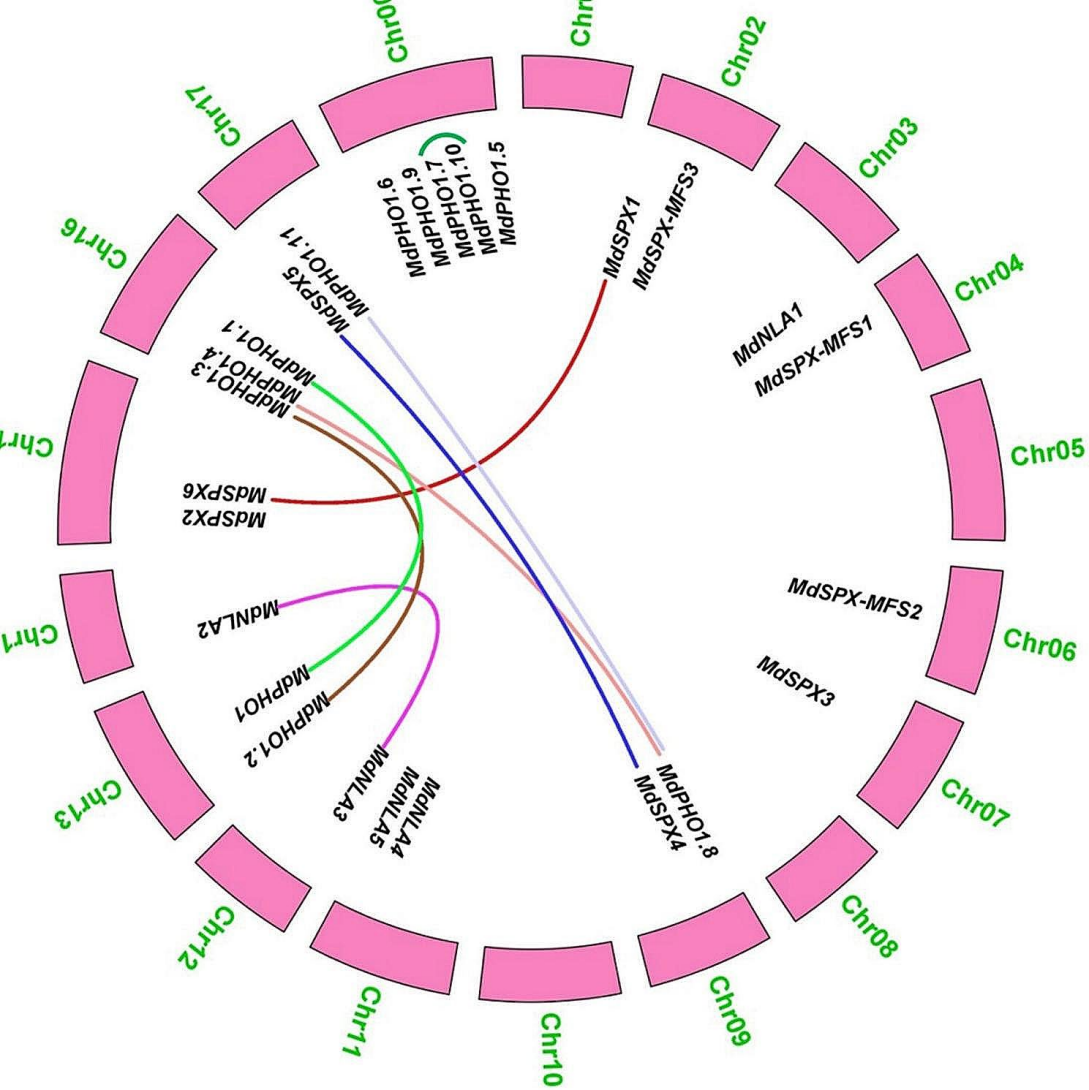
Analysis of gene duplications between chromosomes for the *MdSPX* genes. Different coloured lines connected segmental duplicated *MdSPX* gene pairs, with a total of seven segmental duplications. Small green lines marked tandemly duplicated gene pair

To assess the genomic distribution and duplication of the *MdSPX* genes, we analysed the syntenic regions of the *MdSPX* genes using MCscanX software to examine the duplication of *MdSPXs*. As shown in Table S2, 2814 tandemly duplicated gene pairs and 11,473 segmental duplication blocks were found in the apple genome, respectively. Only one tandemly duplicated gene pair (*MdPHO1.7/MD00G1081300* and *MdPHO1.10/MD00G1081400*) was found in the *MdSPX* gene family (Fig. 4; Table S2). Moreover, we also identified seven pairs of segmental duplication events of *SPX* genes in apple as follows *MdSPX1* and *MdSPX6*; *MdSPX4* and *MdSPX5*; *MdPHO1.8* and *MdPHO1.4*; *MdPHO1* and *MdPHO1.1*; *MdPHO1.2* and *MdPHO1.3*; *MdPHO1.8* and *MdPHO1.11*; *MdNLA2* and *MdNLA3* (Fig. 4; Table S2).

### *Cis*-acting regulatory analysis of *MdSPX* genes

**Fig. 5:**
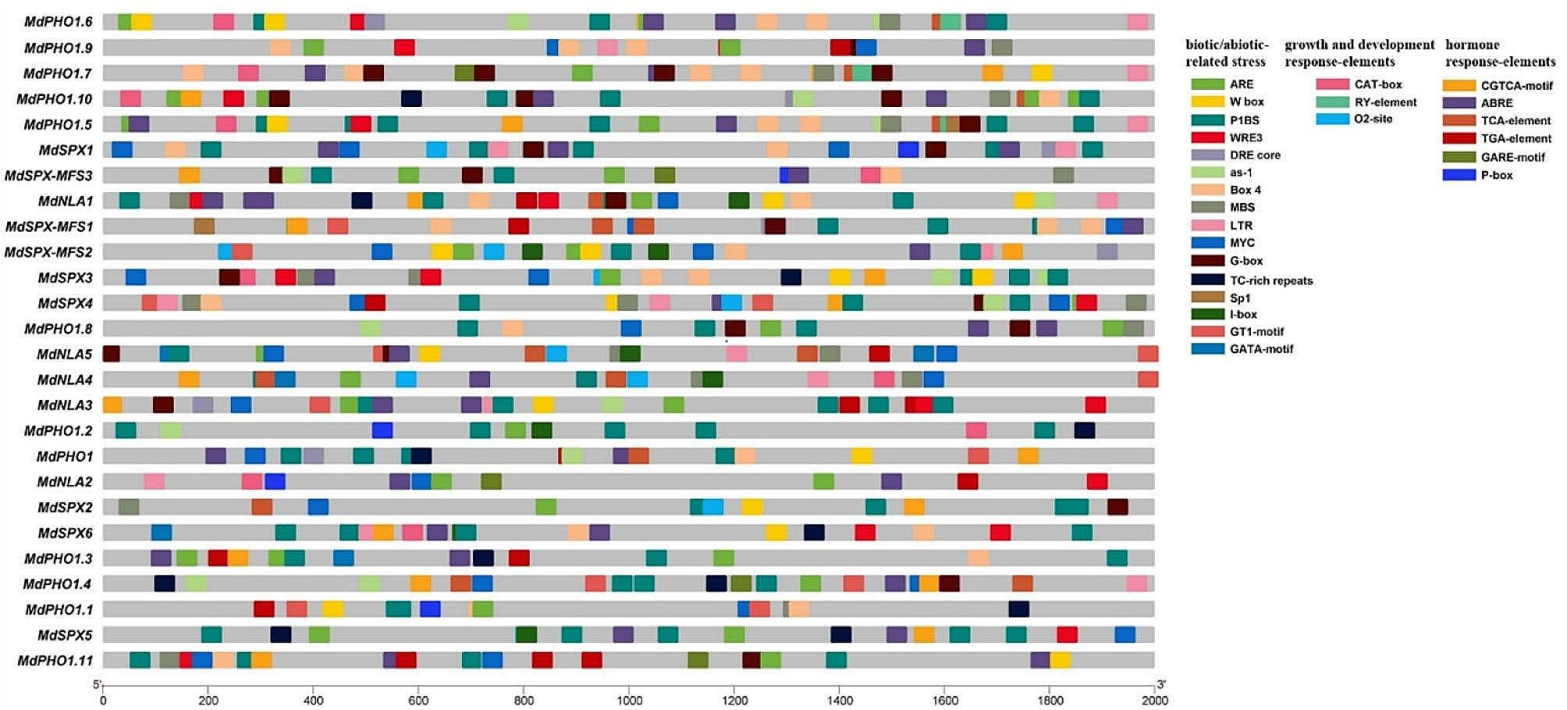
Display of *cis*-elements in the promoters of the *MdSPX* genes in apple. The different colours represented the corresponding *cis*-element types in the *MdSPX* promoters and were classified into 3 main categories: biotic/abiotic-related stress, growth and development response-elements, and hormone response-elements

*Cis*-elements in promoters of genes play an essential role in the overall regulation of gene expression. There is increasing evidence that genes with similar expression patterns may share the same *cis*-elements. The *cis*-elements in the 2-kb upstream of the transcription start site of *MdSPX* genes were predicted using PlantCARE and PlantPAN 3.0 software (Table S3), and three main categories were classified, namely, biotic/abiotic-related stress, growth and development response-elements, and hormone response-elements (Fig. 5; Table S3). Biotic/abiotic-related stresses included light response elements (G-box, GT1-motif, I-box, Box4, GATA-motif, ARE and Sp1), low temperature response element (LTR), drought response elements (MBS, MYC, DRE core and TC-rich repeats) and damage response element (WRE3). We also found that W box elements were extensively present in several *MdSPX* gene promoters, suggesting that these genes might be regulated by WRKY transcription factors. Development response-elements (such as O2-site, CAT-box and RY-element) were also commonly contained in promoters. In addition, six hormone elements related to phytohormones (such as salicylic acid, methyl jasmonate and abscisic acid) were recognised. *SPX* genes play key roles in Pi homeostasis, and the P1BS elements (PHR1 binding site) in their promoters are essential in responding to Pi starvation. P1BS elements were notably enriched in the promoters of all *MdSPX* genes except *MdPHO1.9* and *MdPHO1.7* and multiple copies of P1BS elements were detected in these gene promoters, implying that these genes might be regulated by MdPHR1 under Pi deficiency.

### Expression of *MdSPX* genes under low pi condition

**Fig. 6:**
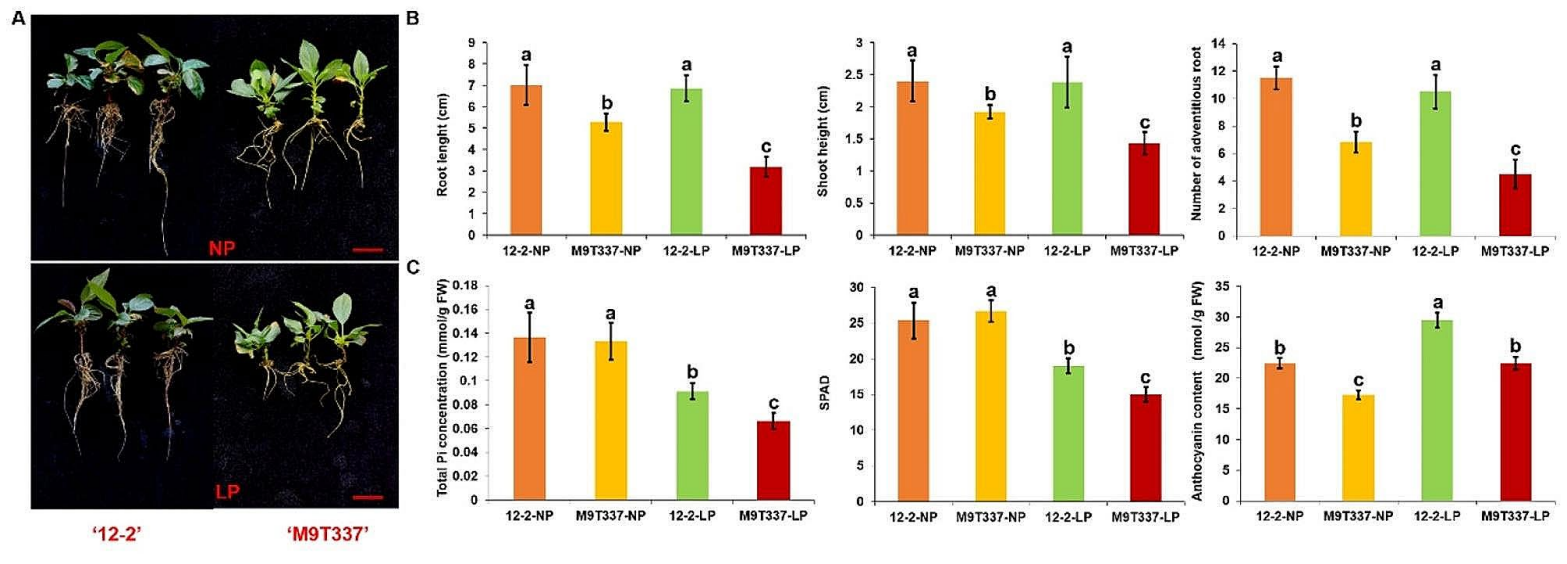
Phenotypic differences between ‘12 − 2’ and ‘M9T337’ plants under phosphorus starvation. (**A**) Phenotypic observations of ‘12 − 2’ and ‘M9T337’ after 21 days of normal (NP: 1 mmol) and phosphorus deficiency stress (LP: 10 µmol). Scale bars = 1 cm. (**B**) The root length, shoot height, and the number of adventitious root between ‘12 − 2’ and ‘M9T337’ plants were measured on the 21 days after normal Pi and Pi deficiency stress treatments. (**C**) The total Pi concentration, SPAD values, and anthocyanin levels between ‘12 − 2’ and ‘M9T337’ plants were measured on the 21 days after normal Pi and Pi deficiency stress treatments. Samples with different letters are significantly different: *P* < 0.05 (Fisher’s LSD mean separation test)

**Fig. 7:**
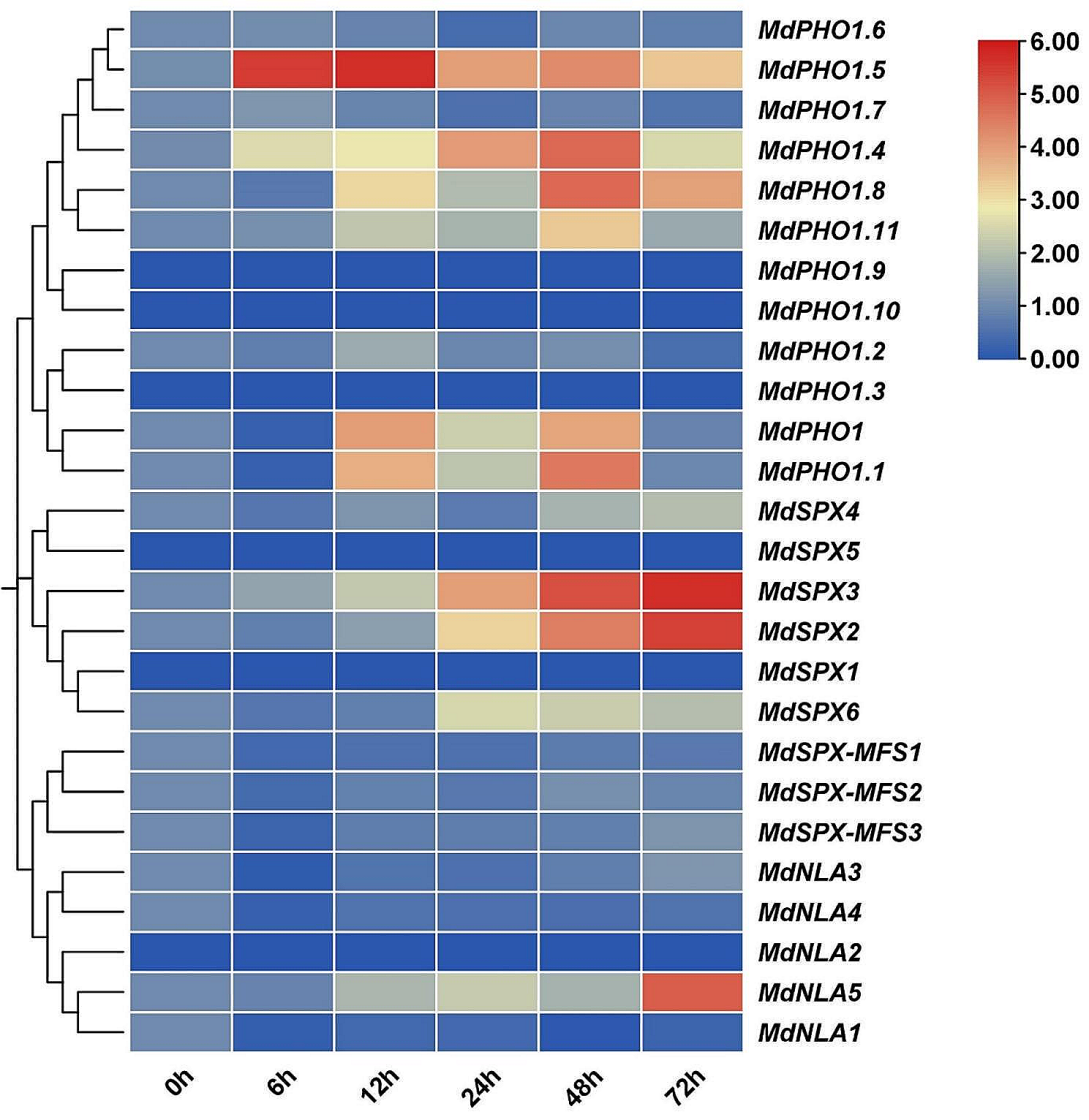
Expression pattern analysis of 26 *MdSPX* genes in ‘12 − 2’ samples at different times of low Pi treatment (10 µmol). Total RNA was extracted from treated roots. The apple *18 S* gene was used as an internal control, and each experiment included three biological replicates

In this study, the self-rooted apple stock superior ‘12 − 2’ and ‘M9T337’ were screened for low Pi stress treatments. The results of our hydroponic experiments showed that the length of adventitious root, shoot height, and the number of adventitious root in ‘M9T337’ changed significantly under low Pi stress treatments but exhibited no significant changes in ‘12 − 2’ plants (Fig. 6A, B). The total Pi concentration, SPAD values, and anthocyanin levels in ‘12 − 2’ were higher than that in ‘M9T337’ under low-Pi conditions (Fig. 6C), indicating that ‘M9T337’ was a Pi-sensitive genotype and that ‘12 − 2’ was a Pi-tolerant genotype. The expression patterns provide essential clues for understanding the regulatory role of *SPX* genes. A number of *MdSPX* family genes were highly induced under low Pi stress condition, among which the expression levels of *MdSPX2*, *MdSPX3*, and *MdPHO1.5* genes showed strong up-regulation in response to low Pi stress (Fig. 7).

### Prediction of the MdSPX protein interaction network

**Fig. 8:**
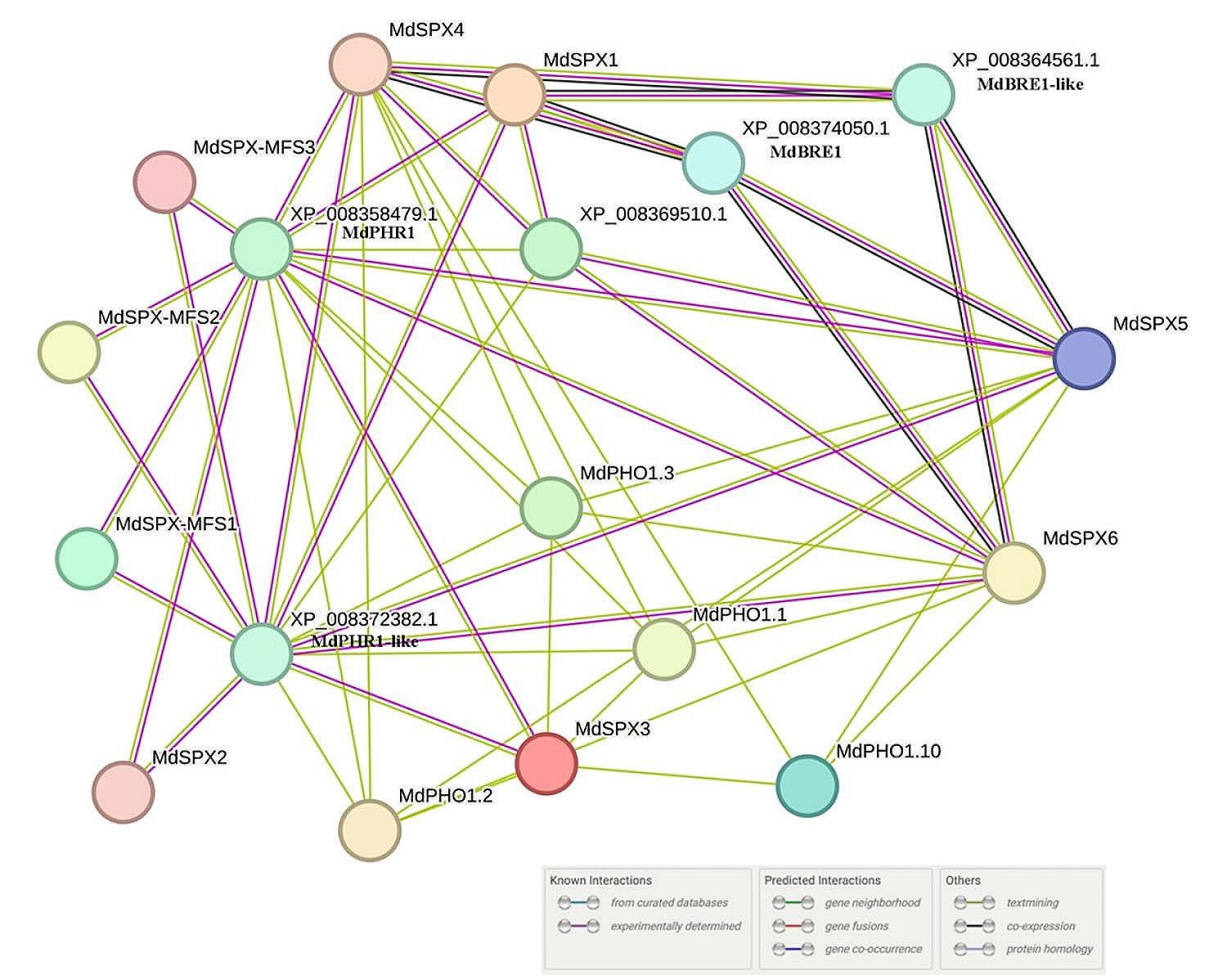
Protein interaction network of MdSPXs. The network was predicted by the online software STRING. The different coloured lines represented the different types of evidence for the prediction of the interaction network

The analysis of protein interaction network has proven to be an effective approach to study gene function. The STRING online software was used to predict the protein interaction network queried with 26 MdSPX protein sequences. MdSPX protein interaction network analysis showed that several MdSPX proteins interacted with each other, for example MdPHO1.3 might interact with MdSPX3, MdSPX4, MdSPX5 and MdSPX6 (Fig. 8). As an important transcription factor in plant phosphorus regulatory network, PHR protein plays a key role in signal transduction regulation under Pi starvation induction. PHR1 normally interacts with proteins containing the SPX domains in a Pi-dependent manner to regulate transcription. We found that phosphate starvation response (MdPHR1 and MdPHR1-like) proteins interacted with several MdSPX proteins, further highlighting the importance of SPX in maintaining phosphorus homeostasis. MdBRE1 and MdBRE1-like, the E3 ubiquitin pathway proteins, were also identified to interact with several MdSPX proteins (Fig. 8). In addition, the deoxyribodipyrimidine photo-lyase (XP_008369510.1), an enzyme that catalyzes chemical reactions, was present in the MdSPX protein interaction network. The predicted protein association network provides a meaningful reference for further studies.

### MdSPXs physically interact with MdPHR1

**Fig. 9:**
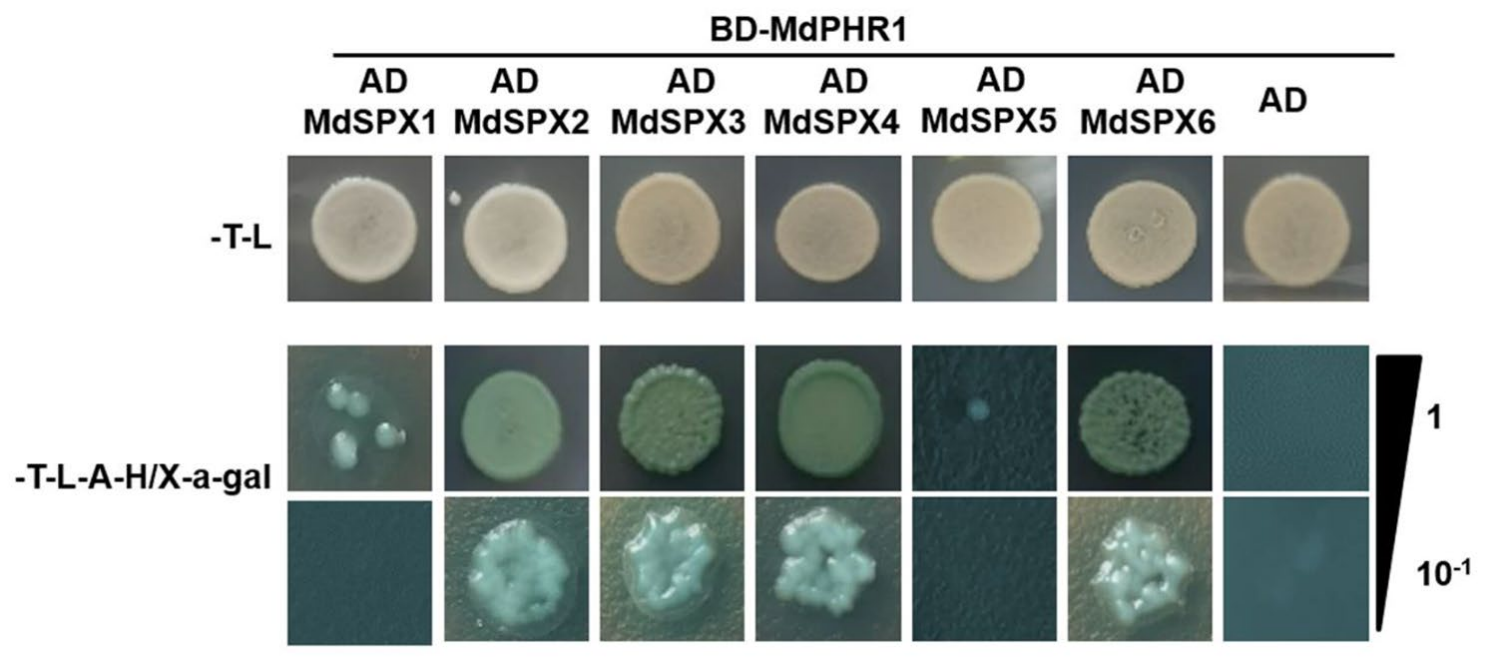
Yeast-two-hybrid assays validating the interaction of MdPHR1 with the MdSPX proteins. SD, Synthetic defined; X-a-Gal, 5-bromo-4-chloro-3-indolyl-a-D-galactopyranoside

The protein interaction network revealed that MdPHR1 interacted with several MdSPX proteins. To verify whether MdPHR1 interacted with MdSPXs, yeast two-hybrid experiments were carried out. The recombinant plasmids AD-MdSPX1, AD-MdSPX2, AD-MdSPX3, AD-MdSPX4, AD-MdSPX5, and AD-MdSPX6 were co-transformed with BD-MdPHR1 in yeast cells, and yeast cells was observed on SD/-Trp-Leu-His-Ade medium with X-a-gal. As shown in Fig. 9, MdPHR1 was found to interact with MdSPX2, MdSPX3, MdSPX4, and MdSPX6, but not with MdSPX1, MdSPX5. To demonstrate biological significance of MdSPX and MdPHR1 interaction, we found that the expression levels of *MdSPX2*, *MdSPX3*, *MdPSI1*, and *MdPSI2* in ‘12 − 2’ were higher than that in ‘M9T337’ in response to low Pi stress (Fig. S2), indicating that these genes might play key role in the Pi-signaling pathway in self-rooted apple stock.

## Discussion

Physiological and metabolic processes in response to phosphorus deficiency in plants depend on a complex and fine-grained phosphorus signalling regulatory network in their bodies [27]. With the development of molecular biology research techniques, more and more transcription factors and phosphorus starvation-responsive genes in plant phosphorus signalling regulatory pathway have been identified, which in turn has greatly contributed to the understanding of the phosphorus signalling network. Genes of the *SPX* domain-containing family are widely involved in the regulation of phosphorus signalling networks. Over the past years, the role of *SPX* family genes in regulating the Pi signal network has been studied in *Arabidopsis*, wheat, rice and maize. However, functional and evolutionary research on *MdSPXs* has been less reported. In this study, we identified a total of 26 *MdSPX* genes and classified them into four subfamilies (Fig. 2; Table 1). Gene structure and motif composition can provide valuable information on the genetic relationships of multi-gene families [28, 29]. As shown in Fig. 2, *SPXs* shared similar gene structure and motif composition within the same group. Similar functions are usually shared by genes with similar structures and conserved motifs. MdSPX proteins were clustered into several *Arabidopsis* functional clades, offering useful guidance for understanding *MdSPX* gene function.

To explore the evolutionary relationship of *MdSPX* family genes with *SPX* genes from other species, we constructed a phylogenetic tree of 157 SPX proteins across six species and broadly grouped them into four subfamilies (Fig. 3). The apple *SPX* family genes were clearly classified into different evolutionary clades compared to the *SPX* family genes in *Triticum aestivum*, *Zea mays*, and *Oryza Sativa*, and the evolutionary relationships of the *SPX-EXS* and *SPX-MFS* subfamily members varied markedly among species, suggesting that these two subfamily proteins may have different roles in different plants. Gene duplication events have been shown to play an important role in the speedy expansion and evolution of gene families. Overall, 2814 tandem duplication gene pairs and 11,473 segmental duplication blocks were observed in apple genome (Table S2). In this study, only one segmental duplication events and seven tandem duplication gene pairs were identified in the *MdSPX* family by MCScanX (Fig. 4; Table S2).

Similar to other species, the *MdSPX* gene promoters included numerous biotic/abiotic-related and hormone-response elements [17]. It has been shown that WRKY transcription factors can regulate the expression of *SPX* members. For example, both WRKY6 and WRKY42 can regulate the transcription of the *AtPHO1* gene by binding to the W-box element [30]. We identified a total of 18 *MdSPX* genes with W-box elements in their promoter regions, indicating that these genes may be regulated by WRKY transcription factor (Fig. 5; Table S3). P1BS (GNATATNC) is a known *cis*-acting element in response to Pi deficiency and clusters at the promoters of a lot of *PSR* genes [27, 31, 32]. For example, 18 *ZmSPX* genes contain P1BS elements in their promoter regions and 31 *BnaSPX* genes contain P1BS elements in their promoter regions [16, 18]. In our results, except for *MdPHO1.9* and *MdPHO1.7*, the remaining 24 *MdSPX* genes contained different numbers of P1BS in their promoter regions (Fig. 5), predicting that the expression of these *SPX* genes in apple could be similarly induced by Pi deficiency. The position, number, and flanking sequence of all elements within a promoter region directly affects promoter activity. The enrichment of different promoter elements suggests that *MdSPXs* might be regulated by a series of related transcription factors.

Analysis of *MdSPXs* expression under different periods of low Pi treatment showed that several *MdSPX* genes were in response to Pi stress, with different expression patterns in the self-rooted apple stock superior ‘12 − 2’ (Fig. 7). Many studies have shown that some *SPX* subfamily genes are up-regulated under low Pi, for instance *AtSPX* and *AtSPX2* [33], and we found that most *MdSPX* subfamily genes, except MdSPX1 and MdSPX5, were significantly induced in response to low Pi in apple, a result consistent with earlier studies [13, 34]. *MdSPX2* and *MdSPX3* were significantly expressed in both ‘12 − 2’ and ‘M9T337’ plants under low Pi stress condition, but the expression levels of *MdSPX2* and *MdSPX3* in ‘12 − 2’ under Pi deficient condition were significantly higher than that in ‘M9T337’, which further suggests the important role of the *SPX* genes in response to low Pi stress.

We also predicted the protein interaction networks of the 26 MdSPX protein sequences queried by STRING online software, which showed that multiple MdSPXs might interact with MdPHR1. AtPHR1 participates in Pi homeostasis by interacting with SPX structural domain-containing proteins at low Pi levels [33]. The results of the Y2H assay showed that MdSPX2, MdSPX3, MdSPX4, and MdSPX6 interacted with MdPHR1, while MdSPX1 and MdSPX5 did not interact with MdPHR1, a result similar to that of maize and rice. These findings suggest that a relatively conserved regulatory network for phosphorus stress response exists in self-rooted apple stock.

## Conclusions

In the apple genome, 26 *MdSPX* genes were identified and divided into four subfamilies. To fully understand the biological features of these *MdSPX* genes, gene structures, conserved motifs, gene duplications, phylogenetic relationships and *cis*-elements were analysed in detail. The results of the expression of all 26 *MdSPX* genes at different times of Pi stress showed that some *MdSPXs* play key roles in Pi deficient response, especially *MdSPX2*, *MdSPX3*, and *MdPHO1.5*, which were highly responsive to low Pi stress. Some members might function under low Pi by interacting with MdPHR1. In conclusion, these results improved the understanding of the apple *SPX* gene family and contribute to future biological studies of *MdSPX* genes in self-rooted apple stock.

## Methods

### Identification of *MdSPX* genes in apple

*MdSPX* gene family members were identified by Blastp search in *Malus×domestica* reference genome database using 20 *Arabidopsis* SPX proteins as query sequences. Additionally, the Hidden Markov Model (HMM) SPX domain (PF03105) in the Pfam database (http://pfam.xfam.org/) were used to search for *MdSPX* genes. All candidate genes were then further verified using the Pfam and SMART (http://smart.embl-heidelberg.de). Twenty-six *MdSPX* genes were finally identified in the apple genome and named in accordance with their phylogenetic relationship to *Arabidopsis SPX* genes.

### Multiple sequence alignment and phylogenetic analysis

To investigate the evolutionary relationships of SPX proteins in *Arabidopsis thaliana*, *Solanum lycopersicum*, *Zea mays*, *Oryza sativa*, *Malus domestica* and *Triticum aestivum*, multiple alignments of 20 AtSPXs, 18 SlSPXs, 32 ZmSPXs, 15 OsSPXs, 26 MdSPXs, and 46 TaSPXs were performed by CLUSTALW, and the obtained results were applied to generate a phylogenetic tree via the neighbour-joining (NJ) method with 1000 bootstrap replicates in MEGA 7. The phylogenetic tree was landscaped using the EvolView tool (http://www.evolgenius.info). It was then classified into four subfamilies based on *SPX* gene family characteristics.

### Analysis of gene structure and conserved motifs

The DNA and cDNA sequences of all *MdSPX* and *AtSPX* genes were extracted from the *Malus domestica* and *Arabidopsis* genomes, respectively, and then the structures of the *SPX* genes were characterised using the Gene Structure Display Server (GSDS: http://gsds.cbi.pku.edu.cn/). The MEME motif discovery tool (http://meme-suite.org/index.html*)* was used for identifying the conserved motif structures of all MdSPX and AtSPX amino acid sequences. Visualization of the results was performed using TBtools.

### Physicochemical properties and chromosome location analysis

The 26 MdSPX protein sequences were examined using the online website ExPASy (http://web.expasy.org/protparam/) to predict their amino acid lengths, molecular weights and isoelectric points (pI) [35]. Chromosome position information of apple was downloaded from the GDR database (https://www.rosaceae.org/). The 26 *MdSPX* genes were mapped to their corresponding chromosomes using Mapchart 2.32 software.

### Gene duplication analysis

Each of the *MdSPX* genes was mapped to the apple chromosome using TBtools, based on physical positional data from the apple genomic database. The Multiple Collinearity Scan toolkit (MCScanX) was used with default parameters to score gene duplication events [36, 37].

### Identification of *cis*-elements in the promoters of the *MdSPX* genes

We extracted 2-kb sequences upstream of the transcription start site of the 26 *MdSPX* genes from the *Malus domestica* database using the TBtools. All extracted target sequences were then uploaded to PlantCARE (http://bioinformatics.psb.ugent.be/webtools/plantcare/html/) and PlantPan3 databases (http://PlantPAN.itps.ncku.edu.tw) for *cis*-element analysis. The obtained data were then preliminarily collated and visualised using Tbtools [38].

### Plant material, pi stress treatments, and qRT-PCR analysis

The deep-rooted ‘12 − 2’ (hybrid seedlings of Ralls and *Malus spectabilis*) self-rooted apple stock and ‘M9T337’ stock, an apple dwarf rootstock were screened. The seedlings of *M. spectabilis* were obtained from the Beijing Botanical Garden (Beijing, China) and those of Ralls and ‘M9T337’ stock were obtained from Shandong Institute of Pomology. The roots of ‘12 − 2’ were used for qRT-PCR analysis. The growth conditions used were those described by [39]. Modified Hoagland medium was used in self-rooted apple stock hydroponic cultivation as described by [18]. An RNAprep pure plant kit (Vazyme, Nanjing, China) was used to isolate total RNA from the samples according to the producer’s instructions. Reverse transcription was then performed with a *EasyScript®* One-Step gDNA Removal and cDNA Synthesis SuperMix (Trans, Beijing, China). Applied Biosystems 7500 real-time PCR system (Applied Biosystems) was used to perform qRT-PCR reactions using UltraSYBR Mixture (with ROX I; Cwbiotech). The results were standardized with apple *18 S* gene. Three biological replicates were used for each test. Table S4 showed the primers used in this study.

### Prediction of protein association networks by STRING

The 26 MdSPX amino acid sequences were uploaded to the online STRING software (http://string-db.org; version 11.5), with the organism selected as " *Malus domestica* “. The highest scoring proteins were selected to construct the network after BLAST analysis. The MdSPXs not interacting with other proteins were deleted. Functional annotations were manually copied from BLAST results. The proteins MdPHR1 (XP_008358479.1), MdPHR1-like (XP_008372382.1), MdBRE1 (XP_008374050.1), and MdBRE1-like (XP_008364561.1) were named based on BLAST results.

### Measurement of the total pi concentration and physiological characteristics

To investigate the Pi sensitivity of ‘12 − 2’ and ‘M9T337’, we measured the root length, shoot height, and the number of adventitious root of the plants on the 21st day after Pi stress treatments. The chlorophyll content was quantified with a SPAD system [18]. The measurement of the total Pi concentration was performed as described by [40].

### Yeast two-hybrid (Y2H) assays

The full-length MdPHR1 CDS was cloned into the bait vector pGBKT7 and the MdSPX1, MdSPX2, MdSPX3, MdSPX4, MdSPX5, and MdSPX6 CDSs were cloned into the prey vector pGADT7 to generate recombinant plasmid AD-MdSPX1, AD-MdSPX2, AD-MdSPX3, AD-MdSPX4, AD-MdSPX5, and AD-MdSPX6. Y2H assays were performed using yeast strain AH109 (Clontech) according to the manufacturer’s instructions. The yeast cells were plated on medium lacking Trp and Leu (SD/-Trp-Leu) and cultured at 28 °C. For interaction screening, the colonies were transferred into medium lacking Trp, Leu, His and adenine (SD/-Trp-Leu-His-Ade) with X-a-gal. Empty vector pGADT7 was used as negative controls.

### Statistical analysis

In this study, the error bars represented the standard error (SE) from at least three biological replicates. The analysis of statistical significance was performed with the student’s t-test at *P* < 0.05 as described [41].

### Electronic supplementary material

Below is the link to the electronic supplementary material.

#### Additional file 1

Supplementary Fig. S1 to S2. (PDF 307 kb)

#### Additional file 2

Table S1 Grouping of SPX proteins. (XLSX 10.2 kb)

#### Additional file 3

Table S2 All tandem duplication and segmental duplication in apple genome. (XLSX 297 kb)

#### Additional file 4

Table S3*Cis*-element analysis of *MdSPX* genes. (XLSX 21.6 kb)

#### Additional file 5

Table S4 The primers used in this study. (XLSX 16.0 kb)

Supplementary Material 1

Supplementary Material 2

Supplementary Material 3

Supplementary Material 4

Supplementary Material 5

## Data Availability

The relevant materials are available from the corresponding authors on reasonable request. The links in this study for the data analyzed are as follows: Pfam database (http://pfam.xfam.org/); SMART (http://smart.embl-heidelberg.de); EvolView tool (http://www.evolgenius.info); Gene Structure Display Server (GSDS: http://gsds.cbi.pku.edu.cn/); MEME motif discovery tool (http://meme-suite.org/index.html); ExPASy (http://web.expasy.org/protparam/); GDR database (https://www.rosaceae.org/); PlantCARE (http://bioinformatics.psb.ugent.be/webtools/plantcare/html/); PlantPan3 databases (http://PlantPAN.itps.ncku.edu.tw); STRING software (http://string-db.org; version 11.5).

## Abbreviations

Pi: Phosphate
At: *Arabidopsis thaliana*
Md: *Malus domestica*
qRT-PCR: quantitative reverse transcription-PCR
CDS: Coding Sequence
PHR1: PHOSPHATE RESPONSE 1

## References

[1] Nussaume L, Kanno S, Javot H, Marin E, Pochon N, Ayadi A, Nakanishi TM, Thibaud MC (2011). Phosphate import in plants: focus on the PHT1 transporters. Front Plant Sci.

[2] Cong WF, Suriyagoda LDB, Lambers H (2020). Tightening the Phosphorus cycle through phosphorus-efficient crop genotypes. Trends Plant Sci.

[3] Fu XZ, Xing F, Wang NQ, Peng LZ, Chun CP, Cao L, Ling LL, Jiang CL (2014). Exogenous spermine pretreatment confers tolerance to combined high-temperature and drought stress in vitro in trifoliate orange seedlings via modulation of antioxidative capacity and expression of stress-related genes. Biotechnol Biotechnol Equip.

[4] Ham BK, Chen J, Yan Y, Lucas WJ (2018). Insights into plant phosphate sensing and signaling. Curr Opin Biotechnol.

[5] López-Arredondo DL, Leyva-González MA, González-Morales SI, López-Bucio J, Herrera-Estrella L (2014). Phosphate nutrition: improving low-phosphate tolerance in crops. Annu Rev Plant Biol.

[6] Yan Z, Chen S, Li J, Alva A, Chen Q (2016). Manure and nitrogen application enhances soil phosphorus mobility in calcareous soil in greenhouses. J Environ Manage.

[7] Zou XH, Wu PF, Chen NL, Wang P, Ma XQ (2015). Chinese fir root response to spatial and temporal heterogeneity of phosphorus availability in the soil. Can J for Res.

[8] Liu N, Shang W, Li C, Jia L, Wang X, Xing G, Zheng W (2018). Evolution of the SPX gene family in plants and its role in the response mechanism to phosphorus stress. Open Biology.

[9] Pipercevic J, Kohl B, Gerasimaite R, Comte-Miserez V, Hostachy S, Müntener T, Agustoni E, Jessen HJ, Fiedler D, Mayer A, Hiller S (2023). Inositol pyrophosphates activate the vacuolar transport chaperone complex in yeast by disrupting a homotypic SPX domain interaction. Nat Commun.

[10] Chiou TJ, Lin SI (2011). Signaling network in sensing phosphate availability in plants. Annu Rev Plant Biol.

[11] Yang J, Wang L, Mao C, Lin H (2017). Characterization of the rice NLA family reveals a key role for OsNLA1 in phosphate homeostasis. Rice.

[12] Yue W, Ying Y, Wang C, Zhao Y, Dong C, Whelan J, Shou H (2017). OsNLA1, a RING-type ubiquitin ligase, maintains phosphate homeostasis in Oryza sativa via degradation of phosphate transporters. Plant J.

[13] Duan K, Yi K, Dang L, Huang H, Wu W, Wu P (2008). Characterization of a sub-family of Arabidopsis genes with the SPX domain reveals their diverse functions in plant tolerance to phosphorus starvation. Plant J.

[14] Secco D, Wang C, Arpat BA, Wang Z, Poirier Y, Tyerman SD, Wu P, Shou H, Whelan J (2012). The emerging importance of the SPX domain-containing proteins in phosphate homeostasis. New Phytol.

[15] Yao Z, Tian J, Liao H (2014). Comparative characterization of GmSPX members reveals that GmSPX3 is involved in phosphate homeostasis in soybean. Ann Botany.

[16] Du H, Yang C, Ding G, Shi L, Xu F (2017). Genome-wide identification and characterization of SPX Domain-Containing members and their responses to phosphate Deficiency in Brassica napus. Front Plant Sci.

[17] Kumar A, Sharma M, Gahlaut V, Nagaraju M, Chaudhary S, Kumar A, Tyagi P, Gajula MNVP, Singh KP (2019). Genome-wide identification, characterization, and expression profiling of SPX gene family in wheat. Int J Biol Macromol.

[18] Xiao J, Xie X, Li C, Xing G, Cheng K, Li H, Liu N, Tan J, Zheng W (2021). Identification of SPX family genes in the maize genome and their expression under different phosphate regimes. Plant Physiol Biochem.

[19] Chen J, Han X, Liu L, Yang B, Zhuo R, Yao X (2023). Genome-wide detection of SPX Family and Profiling of CoSPX-MFS3 in regulating low-phosphate stress in Tea-Oil Camellia. Int J Mol Sci.

[20] Luo J, Liu Z, Yan J, Shi W, Ying Y (2023). Genome-wide identification of SPX Family genes and functional characterization of PeSPX6 and PeSPX-MFS2 in response to low phosphorus in Phyllostachys edulis. Plants-Basel.

[21] Rubio V, Linhares F, Solano R, Martín AC, Iglesias J, Leyva A, Paz-Ares J (2001). A conserved MYB transcription factor involved in phosphate starvation signaling both in vascular plants and in unicellular algae. Genes Dev.

[22] Wild R, Gerasimaite R, Jung JY, Truffault V, Pavlovic I, Schmidt A, Saiardi A, Jessen HJ, Poirier Y, Hothorn M, Mayer A (2016). Control of eukaryotic phosphate homeostasis by inositol polyphosphate sensor domains. Science.

[23] Dong J, Ma G, Sui L, Wei M, Satheesh V, Zhang R, Ge S, Li J, Zhang TE, Wittwer C, Jessen HJ, Zhang H, An GY, Chao DY, Liu D, Lei M (2019). Inositol Pyrophosphate InsP8 acts as an Intracellular Phosphate Signal in Arabidopsis. Mol Plant.

[24] Ried MK, Wild R, Zhu J, Pipercevic J, Sturm K, Broger L, Harmel RK, Abriata LA, Hothorn LA, Fiedler D, Hiller S, Hothorn M (2021). Inositol pyrophosphates promote the interaction of SPX domains with the coiled-coil motif of PHR transcription factors to regulate plant phosphate homeostasis. Nat Commun.

[25] Chabert V, Kim GD, Qiu D, Liu G, Michaillat ML, Jamsheer KM, Jessen HJ, Mayer A (2023). Inositol pyrophosphate dynamics reveals control of the yeast phosphate starvation program through 1,5-IP8 and the SPX domain of Pho81. eLife.

[26] Letunic I, Doerks T, Bork P (2009). SMART 6: recent updates and new developments. Nucleic Acids Res.

[27] Wu P, Shou H, Xu G, Lian X (2013). Improvement of phosphorus efficiency in rice on the basis of understanding phosphate signaling and homeostasis. Curr Opin Plant Biol.

[28] Boudet N, Aubourg S, Toffano-Nioche C, Kreis M, Lecharny A (2001). Evolution of intron/exon structure of DEAD helicase family genes in Arabidopsis, Caenorhabditis, and Drosophila. Genome Res.

[29] Babenko VN, Rogozin IB, Mekhedov SL, Koonin EV (2004). Prevalence of intron gain over intron loss in the evolution of paralogous gene families. Nucleic Acids Res.

[30] Stefanovic A, Ribot C, Rouached H, Wang Y, Chong JL, Belbahri L, Delessert S, Poirier Y (2007). Members of the PHO1 gene family show limited functional redundancy in phosphate transfer to the shoot, and are regulated by phosphate deficiency via distinct pathways. Plant J.

[31] Zhou J, Jiao FC, Wu ZC, Li YY, Wang XM, He XW, Zhong WQ, Wu P (2008). OsPHR2 is involved in phosphate-starvation signaling and excessive phosphate accumulation in shoots of plants. Plant Physiol.

[32] Pant BD, Pant P, Erban A, Huhman D, Kopka J, Scheible WR (2015). Identification of primary and secondary metabolites with phosphorus statusdependent abundance in Arabidopsis, and of the transcription factor PHR1 as a major regulator of metabolic changes during phosphorus limitation. Plant Cell Environ.

[33] Puga MI, Mateos I, Charukesi R, Wang Z, Franco-Zorrilla JM, de Lorenzo L, Irigoyen ML, Masiero S, Bustos R, Rodríguez J, Leyva A, Rubio V, Sommer H, Paz-Ares J (2014). SPX1 is a phosphate-dependent inhibitor of phosphate starvation response 1 in Arabidopsis. Proc Natl Acad Sci USA.

[34] Wang Z, Hu H, Huang H, Duan K, Wu Z, Wu P (2009). Regulation of OsSPX1 and OsSPX3 on expression of OsSPX domain genes and Pi-starvation signaling in rice. J Integr Plant Biol.

[35] Krzywinski M, Schein J, Birol I, Connors J, Gascoyne R, Horsman D, Jones SJ, Marra MA (2009). Circos: an information aesthetic for comparative genomics. Genome Res.

[36] Wang Y, Tang H, Debarry JD, Tan X, Li J, Wang X, Lee TH, Jin H, Marler B, Guo H, Kissinger JC, Paterson AH (2012). MCScanX: a toolkit for detection and evolutionary analysis of gene synteny and collinearity. Nucleic Acids Res.

[37] Chen C, Chen H, Zhang Y, Thomas HR, Frank MH, He Y, Xia R (2020). TBtools: an integrative Toolkit developed for interactive analyses of big Biological Data. Mol Plant.

[38] Li J, Wang T, Han J, Ren Z (2020). Genome-wide identification and characterization of cucumber bHLH family genes and the functional characterization of CsbHLH041 in NaCl and ABA tolerance in Arabidopsis and cucumber. BMC Plant Biol.

[39] Wang Z, Li J, Yang X, Hu Y, Yin Y, Shen X (2022). MdFLP enhances drought tolerance by regulating MdNAC019 in self-rooted apple stocks. Plant Sci.

[40] Zhao H, Wu Y, Shen L, Hou Q, Wu R, Li Z, Deng L, Wen X (2022). Cross-talk between transcriptome analysis and physiological characterization identifies the genes in response to the low phosphorus stress in *Malus mandshurica*. Int J Mol Sci.

[41] Li J, Luan Q, Han J, Chen C, Ren Z (2022). CsMYB60 confers enhanced resistance to *Fusarium solani* by increasing Proanthocyanidin Biosynthesis in Cucumber. Phytopathology.

