# Supplementary material for: Evolution of the SPX gene family and its role in the response mechanism to low phosphorus stress in self-rooted apple stock
Source: BMC Genomics. 2024 May 16;25:488. doi: 10.1186/s12864-024-10402-2 (PMC11108120; doi:10.1186/s12864-024-10402-2)
Supplement: Supplementary file 5 — Supplementary Material 5 [file 12864_2024_10402_MOESM5_ESM.pdf]

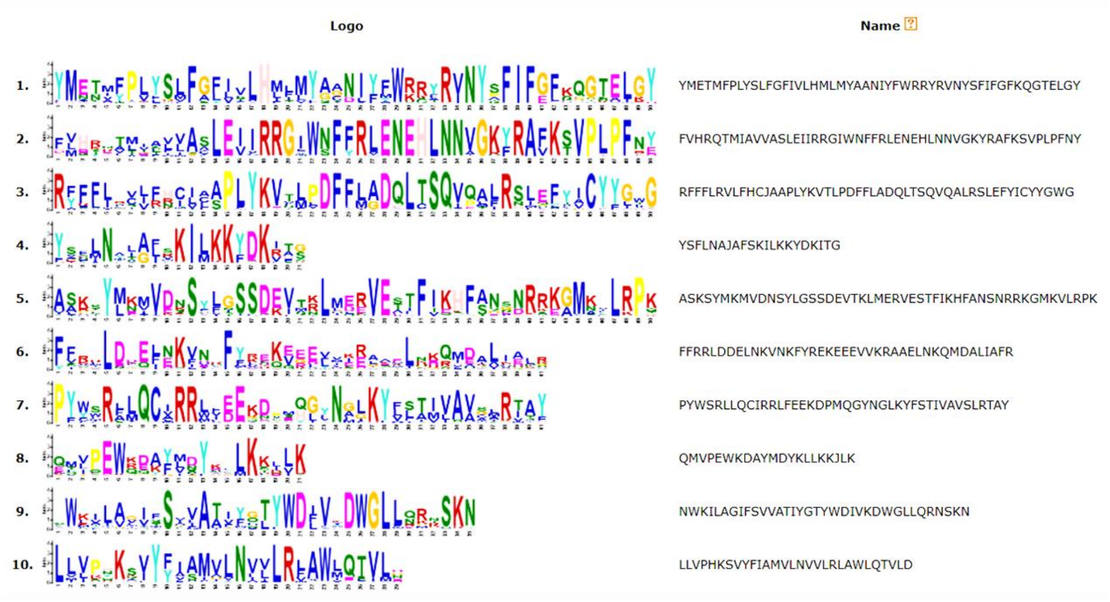

Figure S1. The logos for the 10 conserved motifs of MdSPX proteins derived from the MEME Suite.

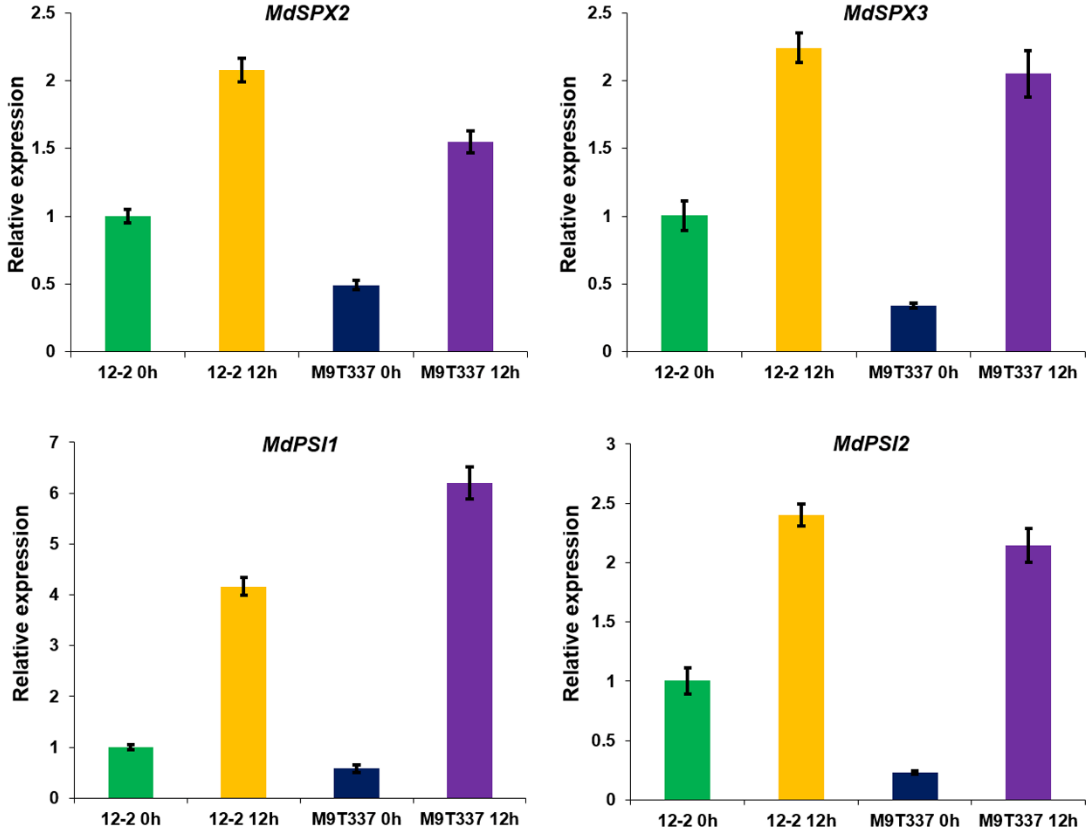

Figure S2. Expression levels of *MdSPX2*, *MdSPX3*, *MdPSI1*, and *MdPSI2* in the roots of ‘12-2’ and ‘M9T337’ after 12 hours of low Pi treatment. Error bars represent ± SE.
